# High-resolution global urban growth projection based on multiple applications of the SLEUTH urban growth model

**DOI:** 10.1038/s41597-019-0048-z

**Published:** 2019-04-18

**Authors:** Yuerong Zhou, Alvin C. G. Varquez, Manabu Kanda

**Affiliations:** 1 Department of Infrastructure Engineering, Melbourne School of Engineering, University of Melbourne, Melbourne, Victoria, Australia; 2 Global Engineering for Development Environment and Society, Department of Transdisciplinary Science and Engineering, School of Environment and Society, Tokyo Institute of Technology, Tokyo, Japan

**Keywords:** Interdisciplinary studies, Environmental social sciences, Developing world, Geography

## Abstract

As urban population is forecast to exceed 60% of the world’s population by 2050, urban growth can be expected. However, research on spatial projections of urban growth at a global scale are limited. We constructed a framework to project global urban growth based on the SLEUTH urban growth model and a database with a resolution of 30 arc-seconds containing urban growth probabilities from 2020 to 2050. Using the historical distribution of the global population from LandScan^TM^ as a proxy for urban land cover, the SLEUTH model was calibrated for the period from 2000 to 2013. This model simulates urban growth using two layers of 50 arc-minutes grids encompassing global urban regions. While varying growth rates are observed in each urban area, the global urban cover is forecast to reach 1.7 × 10^6^ km^2^ by 2050, which is approximately 1.4 times that of the year 2012. A global urban growth database is essential for future environmental planning and assessments, as well as numerical investigations of future urban climates.

## Abstract (summary)

**Table:** 

Design Type(s)	modeling and simulation objective • population modeling objective • time series design • population dynamics analysis objective
Measurement Type(s)	Growth
Technology Type(s)	computational modeling technique
Factor Type(s)	temporal_interval • geographic location
Sample Characteristic(s)	water body • area of protected biodiversity • public road • road • populated place

Machine-accessible metadata file describing the reported data (ISA-Tab format)

## Background & Summary

Global urbanisation has significantly increased over the past few centuries. The United Nations (UN) has projected the global urban population to rise to 68% of the world’s population by 2050, up from the current value of 55%^1^. For studies on climate, environmental resources, ecosystems and urban planning, it is necessary to consider the spatial-temporal changes in urban land cover, especially with regard to the prediction of future urban growth and its impact^2–6^. With rapid advances in remote sensing (RS) and geographical information system (GIS) techniques, a diverse range of models have been developed and improved to simulate and predict future urban growth over the past few decades^7,8^. Among these, cellular automata (CA) have been widely applied. The Clarke Urban Growth Model (UGM), which can estimate two-dimensional urban growth, is one such example^9^.

However, due to the complexity of urbanisation^10^, the urban growth conditions estimated for a region are usually applicable only to that region, where the rate of urbanisation can be assumed to be homogeneous. Thus, past research on future urban expansion scenarios has mostly been limited to a single city^2–4^. Few studies^5^ have attempted to estimate a full global urban growth projection. With the arrival of widely available satellite and GIS products in recent years, coupled by rapid advances in computational capabilities, we developed a global framework for calculating the global urban growth projection by multiple applications of the SLEUTH model (GUGPS).

The framework’s base model is the SLEUTH urban growth model, which is an improvement of the UGM^9^. SLEUTH is an acronym for the spatial inputs used in the model, which are, slope, land cover, excluded regions, urban land cover, transportation, and hill shade. For each model run, five growth parameters are required and can be automatically calibrated, namely *dispersion*, *breed*, *spread*, *slope*, and *road*^4^. Since our purpose was to estimate future urban cover, we excluded the land cover inputs.

The global urban cover was derived from the LandScan^TM^ annual global population distribution datasets from 2000 to 2013^11^ (Oak Ridge National Laboratory https://landscan.ornl.gov/landscan-datasets). Topography, water bodies, the exclusion layer, and transportation networks were extracted from the Global Land 1-km Base Elevation Digital Elevation Model^12^ (GLOBE DEM), the World Water Bodies^13^, the World Database on Protected Areas (WDPA, UNEP-WCMC https://www.protectedplanet.net), and Global Roads Open Access Data Set^14^ (gROADS) supplemented with OpenStreetMap (https://www.openstreetmap.org). The modelling resolution follows that of the LandScan^TM^ datasets, which is that of 30 arc-seconds (~1 km near the equator).

**Fig. 1:**
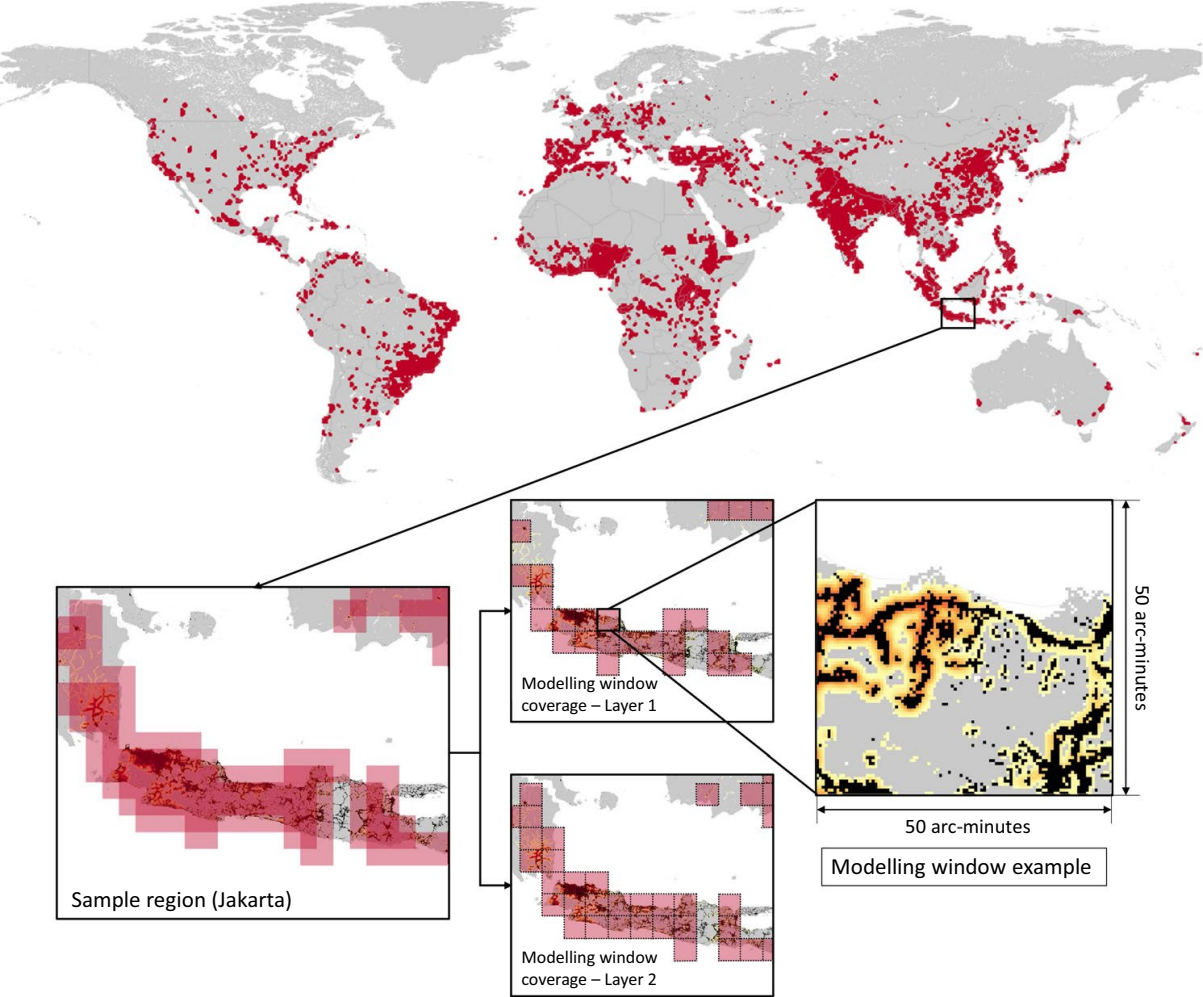
Global coverage of the GUGPS modelling framework and an example of modelling window area. The coordinates refer to WGS 84. The modelling window size is 50 arc-minutes by 50 arc-minutes, and the grid cell in each modelling window is 30 arc-seconds (approximately 1 km at the equator). The example locates on the east of Jakarta, Indonesia.

The framework covered almost all of the urban and rapidly urbanising areas in the world, with two overlapped layers of modelling windows with a resolution of 50 arc-minutes, as shown in Fig. 1. Details related to the criteria used for choosing the modelling area are provided in the sub-section on *Configuring Model Windows and Detecting Potential Urban Growth* in the *Methods* section. As shown in the example in the figure, each modelling window was an individual unit for calibrating and running the SLEUTH model, which allows the framework to detect city-scale variance of urban development. All of the predictions provided by the SLEUTH model in each window were then merged together to obtain the GUGPS results.

The GUGPS dataset contains the future urbanisation projections from 2020 to 2050, with probabilistic results covering all the regions globally. The projection follows the urbanising trends during the calibration period, from 2000 to 2013, which means that future urban growth is assumed to be comparable with the growth of cities during this period. However, future urban growth will be affected by more factors, including but not limited to, social and economic situations, global and regional policies, as well as environmental issues and regulations. Therefore, as basic futuristic urban cover information for global studies on climate change, ecosystems, environmental resources, economic and political decision-making regarding urban regions, the GUGPS dataset can be used. Modifications or improvements may be required during application, especially if the target region experiences significant social or economic events, or when the area of study is located mostly along the boundaries of the modelling windows. This can be achieved by following the framework used in this study and modifying the growth coefficients or surface inputs accordingly.

## Methods

**Fig. 2:**
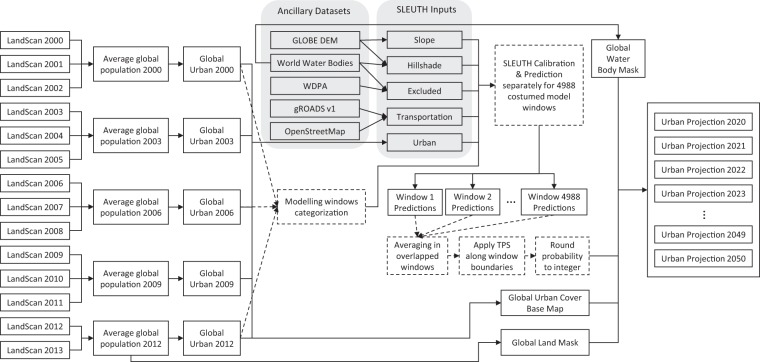
Schematic overview of the workflow adopted to generate GUGPS datasets.

The historical global urban cover maps at spatial resolutions of 30 arc-seconds were produced by defining urban areas based on the LandScan^TM^ ^11^ global population distribution datasets. These urban maps were then used to generate appropriate windows with resolutions of 50 arc-minutes, covering major urban are OpenStreetMap as globally. In each of the windows, the SLEUTH model was calibrated and applied to project future urban growth. Other inputs required by SLEUTH, except for the historical urban cover, were prepared by deriving necessary information from ancillary datasets including the GLOBE DEM^12^, the WDPA, gROADS^14^, as well as OpenStreetMap (refer to “Background and summary” for the description of the acronyms). The urban growth projections in all the modelling windows’ output by the SLEUTH model were combined and, finally, the latest global urban cover, as well as the global land and water masks, were added to form the GUGPS. The workflow is shown in Fig. 2 and described in detail in the following sections.

### Urban definition

Population distribution is a major component of the historical global urban cover estimation. We acquired annual population distributions from LandScan^TM^ ^11^. The population distributions are essentially a combination of locally adaptive models that allocate population counts into gridded areas at a resolution of 30 arc-seconds, based on regional data and geographical conditions^11^. Though the population distribution is available annually, it fluctuates due to improved methods and data conditions. To minimise uncertainty, the population distributions were averaged for every three years, from 2000 to 2011. We acquired representative population distributions from 2000, 2003, 2006 and 2009. We included the distribution for 2012, which comprises the average distributions from 2012 and 2013. The five historical representative population distributions were then used to define global urban distributions. Based on the methodology for defining urban areas used by the Organization for Economic Co-Operation and Development (OECD)^15^, urban areas were identified as 30 × 30 arc-second grids with population densities exceeding 1,000 people per sq. km. Five historical global urban distribution maps were acquired and used as urban cover inputs to SLEUTH.

It is noteworthy that, due to some features of the LandScan^TM^ datasets, the prepared urban maps contain a considerable number of scattered (or discrete) ‘urban’ grids. These ‘false urban detections’ are mainly distributed in small towns and villages, and along traffic networks. We did not exclude these scattered detections from our urban land cover maps because they have urbanising potential due to their population concentrations. However, they should not be considered as actual urban areas, so they were not included when configuring the model windows in the following section.

### Configuring Model Windows and Detecting Potential Urban Growth

The modelling window (refer to “Background and summary” for definition and example) has a resolution of 50 arc-minutes (approximately 10^4^ km^2^), which was based on the derived global urban distribution maps from 2000, 2006 and 2012. Firstly, we aggregated the three global urban maps with a spatial resolution of 50 arc-minutes and determined the total urban area of the window. Windows with less than 25 grids (approximately 25 km^2^) of urban area according to the 2012 global urban map were excluded from the modelling process by SLEUTH. As mentioned at the end of the *Urban Definition* section, ‘false urban detections’ were not expected to contain any considerable urban centres or significant urban growth.

Secondly, as pre-analysis, we conducted a full calibration of each urban agglomeration among the top 31 largest urban agglomerations^16^, and compared the calibrated parameters with the urban growth index (UGI, defined in Equation (1)) of each urban agglomeration. We found that three (d*ispersion*, b*reed*, and s*pread*) out of the five parameters (excluding s*lope* and r*oad*) were calibrated to the minimum value (one) when the UGI fell below 1.5%.

1 $$Urban\,Growth\,Index\,(UGI)\,( \% )=\frac{U{A}_{2012}\,-\,min(U{A}_{2000},\,U{A}_{2006})}{Total\,Area}\times 100 \% $$

Where,

- UA represents the number of grids of urban area in the window in that year (subscript);
- Total Area is the total number of grids in the window;
- min() is the function returning the minimum of the listed variables.

Thus, we calculated the UGIs for each window prior to the global implementation of SLEUTH to verify a plausible amount of change in the urban area within that window. The calculated UGIs were used to determine the calibration mode for each window. For example, when the UGI of the window was less than 1.5%, all three parameters, d*ispersion*, *breed* and s*pread*, were set to 1 during calibration. Furthermore, as the minimum value was 1, the growth coefficients could potentially overestimate areas of extremely low urban growth. Thus, in addition to the 1.5% threshold, model windows with UGIs of less than 0.25% were excluded from the modelling process to avoid overestimating the urban growth.

Therefore, for modelling windows with UGIs between 0.25% and 1.5%, we applied a simplified SLEUTH model calibration mode by setting the values of the three aforementioned parameters to 1. We considered this simplification to be reasonable based on the pre-analysis, which we carried out for large urban agglomerations. The reason for this is that, even if we fully calibrated all five parameters, the three growth parameters would still be calibrated to 1 in these cases. This simplification has little effect on the projected results but provides the added benefit of reducing computational costs.

Following the procedures described above, we defined the first layer of the modelling windows. Full SLEUTH calibration (all five parameters calibrated) was carried out for 416 windows and the simplified SLEUTH calibration (only s*lope* and r*oad* were calibrated, and the other three parameters were fixed at 1) was implemented for the other 2132. We obtained the second layer of modelling windows by shifting the boundaries of the windows by 25 arc-minutes, re-aggregating the global urban maps, and following the procedures described earlier. The processes are summarised in Table 1.

**Table 1:** Numbers of modelled windows in two modelling layers.

Modelling Layer	Layer 1		Layer 2		Memo
Total number of windows with “urban” detected in 2012	10783		10764		Grid with population over 1000/km^2^ exists, including ‘false urban detections’
Number of windows with more than 25 urban grids	5239		5244		Estimated population exceeding 50,000/10^4^ km^2^
Number of windows with UGI Greater Than 0.25%	2548		2516		Above or equal to the minimal growth level defined in SLEUTH
**Calibration Mode**	**Full**	**Simplified**	**Full**	**Simplified**	Full mode: UGI greater than 1.5%, Simplified mode: UGI between 0.25% and 1.5%. Reason for failed cases: not enough detected urban in 2000.
Number of detected windows	416	2132	415	2101	
Number of modelled windows	416	2096	415	2061	
Percentage modelled	100%	98.31%	100%	98.10%	

### SLEUTH Urban Growth Modelling

The inputs to the SLEUTH Urban Growth model include five sets of grayscale gif images, representing the distributions of the terrain slope, excluded areas for urbanisation, historical urban distribution, transportation networks, and hill shade^4^. The urban images were prepared based on the five global urban maps generated from the LandScan^TM^ population datasets. The global urban maps were converted into gif format. All inputs were prepared for each modelling window.

Slope images were obtained by calculating the percentage slope from the GLOBE DEM^12^. The hill shade inputs consist of hill shades and water bodies, which we acquired from the GLOBE DEM and World Water Bodies, respectively. The raster package in R was used to process the DEM into hill shade images, which were further masked by a rasterised water body map and converted into gif format. The rasterised World Water Bodies map and the WDPA were used to prepare the excluded inputs; this prevents urbanisation of water bodies and protected areas.

The transportation images were mainly derived from gROADS^14^. The Line type shapefiles from gROADS were in the World Geodetic System (WGS 84) and were projected into the Universal Transverse Mercator (UTM) coordinate system so that we could calculate the total length of roads within each 1 km grid. We then projected the raster file containing the road lengths back into the WGS 84 datum. The values in the raster were then normalised, weighted, and fitted to integers between 0 and 4. Grids with values of 3 were adjusted to 4 to fit with the required scaling of road densities in SLEUTH. For regions lacking road information in gROADS, the *primary*, *secondary* and *tertiary* classes of roads in OpenStreetMap were used as an alternative and processed using the approach as described earlier. In total, 44 windows used the transportation map prepared from OpenStreetMap.

Finally, we calibrated the results using the two calibration modes (full mode and simplified mode, defined previously), which we classified based on the UGI. All of the windows in the two layers run in full calibration mode were completed successfully. Thirty-six windows from layer 1 (1.69%) and 40 in layer 2 (1.9%), as shown in Table 1, of the runs in simplified calibration mode failed due to insufficient urban area in the image from the year 2000, which we used for modelling. These failed cases were classified as non-urban and exempted from the modelling windows. After calibration, we used SLEUTH to generate our predictions for each window and estimated the predicted annual urban growth scenarios from 2020 to 2050 based on the probability of urbanisation in each grid. The calibration and predictions using SLEUTH were carried out automatically using the *sleuth-automation 1*.*0*.*2* Python package.

## Results Integration

The future urban projections output by SLEUTH in each modelling window from 2020 to 2050 were acquired and converted into geo-referenced tiff format. The final future urbanisation probability of each grid cell was taken from averaging the two probability values estimated from the two global layers. To reduce spatial discontinuity at the window boundaries, we applied a thin plate spline at boundaries where the probabilities differed significantly. The final global urban growth projections from 2020 to 2050 were defined in terms of the urbanisation probability and output in integer format.

## Data Records

The high-resolution GUGPS datasets described in this article, which refer to annual probabilistic maps from 2020 to 2050, are publicly and freely available through Figshare^17^ and at the Kanda Laboratory Repository website (Tokyo Institute of Technology http://urbanclimate.tse.ens.titech.ac.jp/). Each GUGPS dataset represents the projected urbanisation scenario in that year, at a spatial resolution of 30 arc-seconds, in GeoTIFF format. The raster has 102 categories. Categories from 0 to 100 represent the percentage probability of urbanisation. Category 111 refers to the existing urban area in 2012. Water bodies are masked as NA in the raster. Table 2 shows some basic information about the four decade-year GUGPS results.

**Table 2:** Examples of name, description, resolution and format of the decades GUGPS results.

Name	Description	Resolution	Format
global_final_2020.tif	Global urban growth projection in 2020	30 arc-seconds	GeoTIFF
global_final_2021.tif	Global urban growth projection in 2021	30 arc-seconds	GeoTIFF
global_final_2022.tif	Global urban growth projection in 2022	30 arc-seconds	GeoTIFF
global_final_2023.tif	Global urban growth projection in 2023	30 arc-seconds	GeoTIFF
global_final_2024.tif	Global urban growth projection in 2024	30 arc-seconds	GeoTIFF
global_final_2030.tif	Global urban growth projection in 2030	30 arc-seconds	GeoTIFF
global_final_2040.tif	Global urban growth projection in 2040	30 arc-seconds	GeoTIFF
global_final_2050.tif	Global urban growth projection in 2050	30 arc-seconds	GeoTIFF

## Technical Validation

The GUGPS dataset, along with the inputs collected, processed and used for its construction, were verified by the owners. The SLEUTH model has been utilised and validated by developers, researchers and users, and has been applied in many different regions, including but not limited to, cities in Europe^4,18,19^, China^20–23^, United States^24–27^ and the Middle East^3,28,29^.

The urban cover maps defined using LandScan^TM^ were validated by comparing them with the projected global urban cover data estimated by Seto, Güneralp and Hutyra^5^ (Seto’s), which was initialised from a 5-km re-sample of the Moderate Resolution Imaging Spectroradiometer (MODIS) global urban extent^30^. As shown in Table 3, when assuming a static annual growth rate from the year 2000, the projected urban area in 2012 can be estimated to be around 1,169,718 km^2^ based on Seto’s projection. This value is comparable to our estimated input total urban cover, which is 1,268,720 km^2^.

**Table 3:** Comparison of global urban area and growth rates in Seto’s and GUGPS datasets.

Dataset	Seto’s	GUGPS
Resolution	150 arc-seconds	30 arc-seconds
Resolution of simulations (homogeneous region assumption)	Modified UN regions	50 arc-minutes
Start year	2000	2012
2000 urban area (approx. km^2^)	722,225	/
2012 urban area (approx. km^2^)	1,169,718	1,268,720
2030 urban area (approx. km^2^)	2,374,204	1,467,226
2050 urban area (approx. km^2^)	/	1,731,428
Global annual urban area growth rate	4.1%	0.83%

**Fig. 3:**
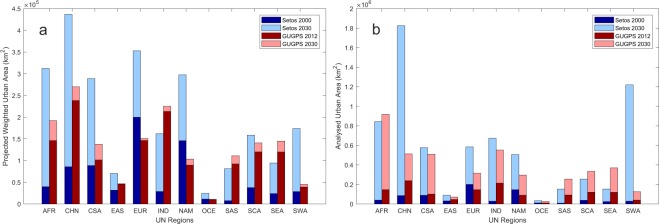
Comparison of projected urban area in different UN regions between Seto’s and GUGPS datasets. (**a**) Total urban area in each region weighted with urbanization probability, (**b**) Total analyzed urban area in each region with urbanization probability greater than 0. Area approximated by assuming each 30 arc-seconds grid as 1 km^2^ area.

The GUGPS dataset for 2030 was verified by comparing it to the Seto’s projections^5,31^. Firstly, the global urbanisation projections were categorised into 16 United Nation (UN) regions^31^ and the statistics of the two datasets within each region were calculated, as shown in Fig. 3. The results of our comparisons indicate that Seto’s team projected more significant urban growth in Africa (AFR), China (CHN), Latin America and the Caribbean (CSA), Europe (EUR), Northern America (NAM), and Southwest Asia (SWA). In EUR and NAM, Seto’s projection predicts that urban areas will increase more by 2030 than predicted by GUGPS. However, Seto’s projection started from a considerably larger urban area, with their area in the year 2000 exceeding the GUGPS urban area projection for 2030.

As EUR and NAM are developed regions, their urban infrastructures are well constructed, but the urban population densities are relatively low compared to those of developing countries. While Seto’s initial urban extent estimation was developed using a satellite-image-based product, our population-based method may underestimate the actual physically constructed area due to the low population density. However, the population growth rate in developed countries is usually lower than that of developing countries^32^, and the expansion of the urban extent of developed cities such as Tokyo is not as significant as that of developing cities, such as Shanghai in China. These factors limit the expansion of urban area in developed regions, which may explain the differences between the two projections.

As the GUGPS dataset classified regions in terms of 50 arc-minutes windows, we were able to model inhomogeneous urban development within countries. The existence of small or slowly developing cities means that the bulk global urban growth rate is expected to be considerably lower than that estimated for developing cities. Angel *et al*.^33^ estimated the urban area growth of developing cities to be 2.5 times that of 2000 by 2030. They predicted a 3.1% annual urban cover growth rate for developing cities by substituting this ratio into Equation (2). As shown in Table 3, the global bulk urban area growth rate of Seto’s projection can be calculated to be 4.1%, whereas the GUGPS dataset suggests a 0.83% annual increase globally.

Though the exclusion of the districts with ‘false urban detections’ was based under the consideration of its historical condition in LandScan^TM^, future anthropogenic interventions such as unforeseen urban planning and development policies could lead to a more rapid urban growth in those districts than the GUGPS suggests. Under these conditions, the GUGPS framework may potentially underestimate future urbanisation of small towns and villages (i.e. model windows with less than 25 km^2^ of urban area).

We further analysed the urban growth rates of the 31 largest urban agglomerations in 2016^16^, the details of which can be found in Table 4, based on Equation (3). These cities were then categorised into two groups based on whether they are in developed or developing countries, and the statistics are summarised in Table 5. Though there are differences between the developed and developing country groups in both datasets, Seto’s projection suggests a higher increase, 4.91%, for cities in developing countries, which significantly exceeds the estimation of 3.1% from Angel *et al*.^33^ for developing cities, while GUGPS has a more conservative prediction, 0.24%, for cities in developed countries.

**Table 4:** Top 31 largest urban agglomerations’ annual urban area growth rates in Seto’s and the GUGPS datasets (Inf in Seto’s group indicates that no original urban area was presented in the year 2000).

Rank	City	Country	Abbreviation	SETO (%)	GUGSP (%)
1	Tokyo	Japan	JPN	2.88	0.15
2	Delhi	India	IND	Inf	0.27
3	Shanghai	China	CHN	6.52	2.53
4	Mumbai (Bombay)	India	IND	4.26	0.11
5	Sao Paulo	Brazil	BRA	3.99	5.68
6	Beijing	China	CHN	4.03	1.69
7	Ciudad de Maxico (Mexico City)	Mexico	MEX	4.68	0.09
8	Kinki MMA (Osaka)	Japan	JPN	2.77	0.13
9	Al Qahirah (Cairo)	Egypt	EGY	7.35	0.36
10	New York Newark	USA	USA	1.86	0.21
11	Dhaka	Bangladesh	BGD	9.95	1.6
12	Karachi	Pakistan	PAK	3.58	0.25
13	Buenos Aires	Argentina	ARG	3.51	1.59
14	Kolkata (Calcutta)	India	IND	9.51	0.25
15	Istanbul	Turkey	TUR	4.25	3.33
16	Chongqing	China	CHN	Inf	0.11
17	Lagos	Nigeria	NGA	7.74	1.42
18	Manila	Philippines	PHL	4.66	0.2
19	Guangzhou, Guangdong	China	CHN	3.74	3.96
20	Rio de Janeiro	Brazil	BRA	2.99	0.12
21	Los Angeles Long Beach Santa Ana	USA	USA	1.22	0.65
22	Moskva (Moscow)	Russian Federation	RUS	1.87	0.13
23	Kinshasa	Democratic Republic of the Congo	COD	4.59	5.42
24	Tianjin	China	CHN	4.28	1.06
25	Paris	France	FRA	Inf	0.15
26	Shenzhen	China	CHN	3.78	4.73
27	Jakarta	Indonesia	IDN	4.74	2.11
28	Bangalore	India	IND	Inf	0.16
29	London	United Kingdom	GBR	4.29	0.29
30	Chennai (Madras)	India	IND	2.3	0.29
31	Lima	Peru	PER	2.61	0.04

**Table 5:** Means and standard deviations of the urban growth rates for the 31 largest urban agglomerations, in developed countries and developing countries, for Seto’s and GUGPS projections, respectively.

Country group	Index	Seto’s (%)	GUGPS (%)
Developed Country	Mean	2.48	0.24
	Standard Deviation	0.99	0.17
Developing Country	Mean	4.91	1.56
	Standard Deviation	2.05	1.77

**Fig. 4:**
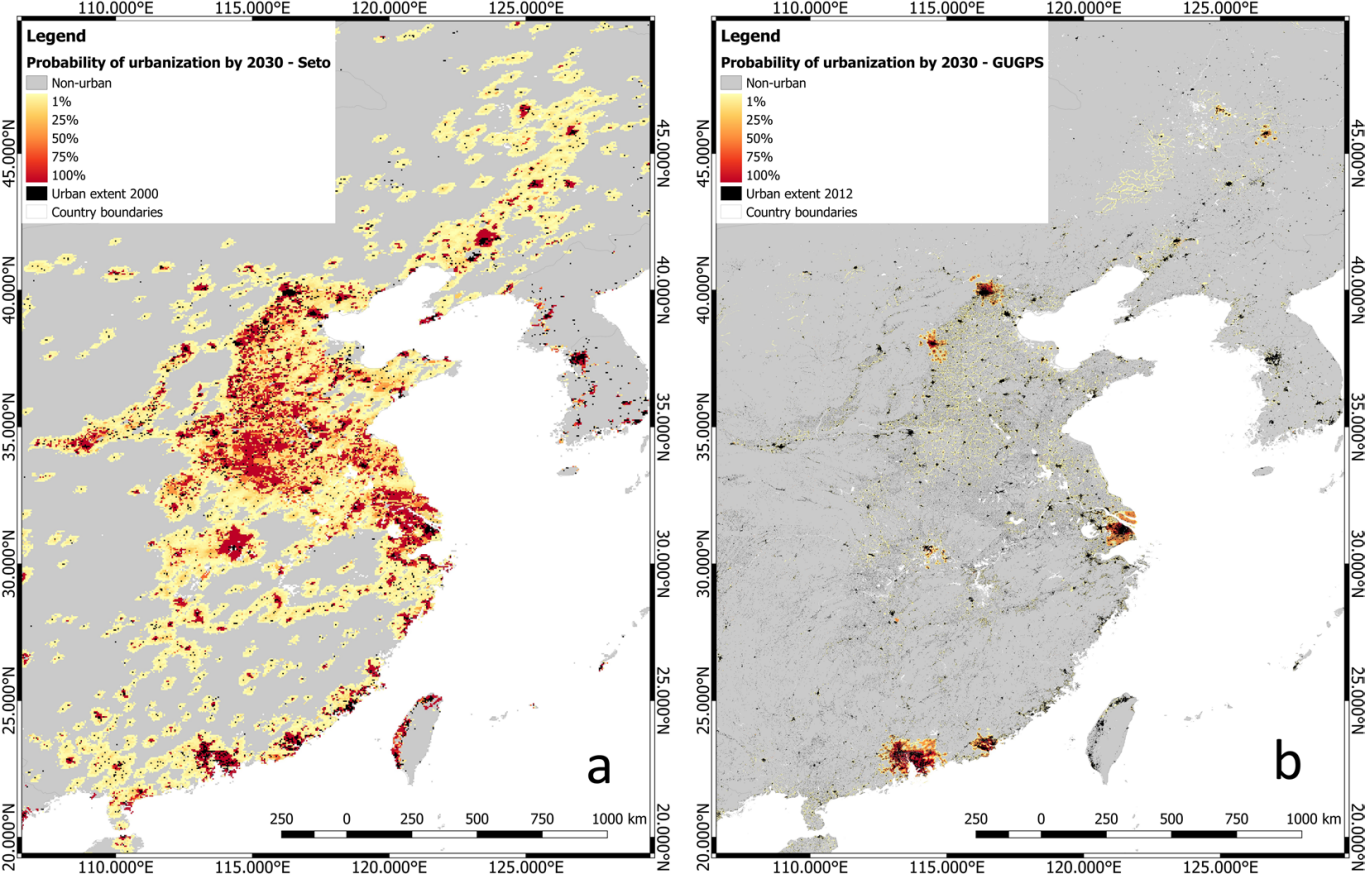
Comparison of urban growth projections by 2030 in the east of China between Seto’s and GUGPS datasets. (**a**) Seto**’**s projection, re-projected to WGS 84 coordinates from Goode**’**s Homolosine Equal-Area projection, original resolution of 5-km, (**b**) GUGPS projection, coordinates refer to WGS 84, resolution of 30 arc-seconds (approximately 1 km at the equator).

Therefore, the differences in urban growth between the two projections for the regions AFR, CHN, CSA and SWA can be explained by the fact that our method allows for inhomogeneous urban development within regions. A typical example is shown in Fig. 4, which shows a comparison between the two datasets’ urban growth projections for the east of China up to 2030. According to the GUGPS dataset, urban development was mainly projected to take place in large cities, including Beijing, Shanghai, Hong Kong, Shenzhen, and Guangzhou, while Seto’s projection suggests a high probability of extensive urbanisation across the east of China.

Figure 3(b) shows the total area analysed; both datasets provide similar results in most regions where the projection suggests that the probability of urbanisation is greater than 0. We then graphically analysed the difference between the CHN and SWA regions and found that it mainly arises for a similar reason to that explained previously, i.e. due to the differences in small cities and underdeveloped areas.

2 $$Yearly\,Urban\,Area\,Growth\,Rate\left( \% \right)=\sqrt[Number\,of\,years]{100 \% +Percent\,Growth}\,-\,100 \% $$

3 $$Urban\,Growth\,Rate/Percent\,Growth( \% )=\,\frac{Urban\,Area\,in\,2030\,-\,Urban\,Area\,in\,Start\,Year}{Urban\,Area\,in\,Start\,Year}\times 100 \% $$

## Usage Notes

We hope that GUGPS datasets will assist researchers studying future urbanisation based on historical evidence from 2000 to 2013. In studies where the impacts of urbanisation should be taken into account, inclusion of the spatial distribution of population and various urban parameters such as regional climate change, future environmental resources and ecosystems, economic and political decision-making, and any other fields affected by urbanisation, will mean that the GUGPS can be used to predict future urban cover scenarios.

The datasets have resolutions of 30 arc-seconds, around 1 km, and provide consecutive predictions of urban growth from 2020 to 2050. If required, the datasets can easily be modified to improve regional performance, such as adjusting by factors related to external information, such as gross domestic product (GDP).

The GUGPS described in this paper is not a comprehensive method for predicting urban planning but, rather, represents the assumed extension of the urban development that occurred during the period 2000 to 2013. The complex structure of urban growth depends on many more predictable and unpredictable factors, which are not considered in this framework, such as the global and regional economy and policy, the impact of climate change, regional land use development regulations, major global and regional events, planning decisions related to transportation networks, etc. We included some major factors, including topography, existing traffic networks, protected areas, water bodies and historical urban developing features when applying the SLEUTH model, but the real story may be much more complicated than this. We hope that the GUGPS will demonstrate the impact of urbanisation based on acceptable assumptions and address the lack of a high-resolution urban growth projection database.

It should be noted that the urban cover defined in the GUGPS is entirely population-based. Hence, the projections reflect the evolution of urban population concentrations rather than physical infrastructure. This could result in underestimations in some developed regions, where population growth is not correlated with the development of urban construction. For applications in areas with infrastructure concerns, we recommend advanced checking and regional modification where needed.

Other limitations of this study include the smoothed areas along the modelling boundaries, based on the assumption that short-term urban growth will not exceed a distance of approximately 100 km (the size of modelling windows). This leads to some rectangular-corner-shaped gradients in rapidly growing cities in the projections after 2040. We strongly recommend checking the GUGPS using GIS software, such as ArcGIS or QGIS, prior to application, especially when using regional or city scale projections after 2040. A recommendation for further improving these regions is to adjust them using external masks to ensure that local information remains reliable.

The GUGPS projections are consistent outputs from the SLEUTH model. Therefore, any analysis of urban development within the GUGPS datasets would not be of significance. To simplify applications, one can use the decade-year projections (for 2020, 2030, 2040 and 2050) or even only one of these four projections (such as 2030 projection).

The framework described in this article can potentially be improved and repeated in the future, based on updated inputs from LandScan^TM^ and other resources. Most of these databases will be regularly renewed by the owner, such as the LandScan^TM^ population distribution, which will be renewed annually. Visit the database’s home website (http://urbanclimate.tse.ens.titech.ac.jp/) for further updates.

## ISA-Tab metadata file

Download metadata file

## Data Availability

The R code developed for preparing the inputs to the SLEUTH model and integrating the modelling results into global maps is publicly and freely available^17^. The code consists of two R programming language scripts (version R 3.4.3; https://www.r-project.org/), which prepare simulation inputs and integrate outputs. The script is internally documented to assist understanding and customisation for further use. We have also shared the modified SLEUTH model, as well as the scenario.jinja file in the Python package sleuth-automation^17^ (version 1.0.2; https://pypi.org/project/sleuth-automation/).
